# Chronic early-life lead exposure sensitizes adolescent rats to cocaine: Role of the dopaminergic system

**DOI:** 10.3389/fnmol.2022.946726

**Published:** 2022-08-24

**Authors:** Damaris Albores-Garcia, Kirstie H. Stansfield, Jennifer L. McGlothan, Zoran Bursac, Tomás R. Guilarte

**Affiliations:** ^1^Brain, Behavior and the Environment Laboratory, Department of Environmental Health Sciences, Robert Stempel College of Public Health and Social Work, Florida International University, Miami, FL, United States; ^2^InVitro Cell Research, LLC, New York, NY, United States; ^3^Department of Biostatistics, Robert Stempel College of Public Health and Social Work, Florida International University, Miami, FL, United States

**Keywords:** dopaminergic system, cocaine, D1 dopamine receptor, lead, mental disorder (disease), substance abuse

## Abstract

Exposure to heavy metals has been associated with psychiatric disorders and recent studies suggest an association between childhood lead (Pb^2+^) intoxication and schizophrenia (SZ). In animal models, Pb^2+^ exposure recapitulates key neuropathological and dopaminergic system alterations present in SZ. Given the high comorbidity of mental disorders such as SZ and substance abuse, coupled with evidence showing that Pb^2+^ exposure affects addiction circuits, we hypothesized that early life Pb^2+^ exposure could sensitize neuronal systems relevant to SZ and substance abuse. To this goal, we examined the effects of chronic developmental Pb^2+^ exposure on the acute locomotor response to cocaine (0, 5, and 15 mg kg^–1^) and behavioral sensitization. We also examined the role of the dopaminergic system in the psychostimulant effects of cocaine, and measured D1-dopamine receptor (D1R) levels in the rat brain using [^3^H]-SCH23390 quantitative receptor autoradiography, as well as the ability of the D1R antagonist SCH23390 to block the cocaine effects on locomotor activation. These studies were performed in male and female rats at different developmental ages consisting of juveniles (postnatal, PN14), early-adolescent (PN28), late adolescent (PN50), and adults (PN120). Our results show that chronic developmental Pb^2+^ exposure increases the acute locomotor response to the higher dose of cocaine in Pb^2+^-exposed male adolescent (PN28 and PN50) rats, and to the lower dose of cocaine in adolescent female rats. No changes in the locomotor activity were detected in adult rats. Behavioral sensitization experiments showed a sustained sensitization in early adolescent Pb^2+^-exposed male but not female rats. The cocaine-induced effects on locomotor activity were abrogated by injection of a D1R antagonist suggesting the involvement of this dopamine receptor subtype. Furthermore, Pb^2+^-induced increases D1R levels in several brain regions were prominent in juveniles and early adolescence but not in late adolescence or in adults. In summary, early chronic developmental Pb^2+^ exposure results in age and sex-dependent effect on the locomotor response to cocaine, suggesting differential susceptibilities to the neurotoxic effects of Pb^2+^ exposure. Our data provides further support to the notion that Pb^2+^ exposure is an environmental risk factor for psychiatric disorders and substance abuse.

## Introduction

Lead (Pb^2+^) exposure continues to be a global public health problem (Tong et al., 2000; Rees and Fuller, 2020). Despite the efforts to reduce Pb^2+^ exposure in the United States, the levels of exposure remain significant in many major urban centers, with blood lead levels (BLL) in children under 5 years of age, above the current CDC level of 5 μg/dL (Raymond and Brown, 2017; Shah et al., 2017). In many other countries of Latin America, the Caribbean, the Middle East, Asia, and Africa there are reports of children with BLL ranging 3.3–43 μg/dL (Tuakuila et al., 2013; Olympio et al., 2017; Carpenter et al., 2019). This is best described in a recent UNICEF report of Pb^2+^-exposed children around the world (Rees and Fuller, 2020). This report estimates that there are approximately 123,000–269,000 infants in the United States with BLL > 10 μg/dL, and a worldwide estimate of children with BLL > 10 μg/dL in the 157–574 million range.

Children and their developing central nervous system are the most sensitive group to the neurotoxic effects of Pb^2+^ exposure (Delgado et al., 2018). Recent studies reported that Pb^2+^ exposure (BLL < 10 μg/dL) contributes to an increased risk of mental and behavioral disorders due to psychoactive substance use (Yoon and Ahn, 2016). The neurotoxic effects of childhood Pb^2+^ exposure also results in behavioral and cognitive disorders, and it has been recently suggested to be a risk factor for mental disorders, including schizophrenia (SZ) (Guilarte, 2004, 2009; Opler et al., 2008; Guilarte et al., 2012; Abazyan et al., 2014; Stansfield et al., 2015), a mental disease that affects 1.1% of the world’s population and approximately 3.5 million people in the United States.

Experimental animal studies have shown that Pb^2+^ exposure results in developmental, molecular, and cellular changes that resemble several key features of SZ pathophysiology (Stansfield et al., 2015). Thus, we used this exposure model as an approach to evaluate if Pb^2+^ exposure contributes to increased sensitivity to drugs of abuse. This is of special interest given the high co-morbidity of SZ and substance abuse (Volkow, 2009; Khokhar et al., 2018). Drug abuse is threefold higher in schizophrenic patients than in healthy controls (Volkow, 2009), and approximately 50% of schizophrenic patients have a substance abuse problem, principally with alcohol, marijuana, or cocaine (Khokhar et al., 2018). This high co-morbidity affects their life quality and contributes to a higher mortality rate (Khokhar et al., 2018).

In the United States, the use of psychostimulant drugs is still a significant public health concern, as it is estimated that around 1.6 million Americans have drug abuse problems (Center for Behavioral Health Statistics and Quality, 2016). Recent studies from our laboratory show that chronic developmental Pb^2+^ exposure increases μ-opiate receptor levels in brain regions implicated with addiction circuits (Albores-Garcia et al., 2021) which could sensitize the animals to the effects of opioids, and other drugs. However, the consequences of these changes in the animal behavior and the underlying mechanisms behind SZ and substance abuse comorbidity, as well as the potential role of Pb^2+^ exposure as a risk factor for both pathologies have not been completely elucidated or investigated. The present study aimed to determine if Pb^2+^-exposed animals have an altered sensitivity to the psychostimulant effects of cocaine and its role on the dopaminergic system.

## Materials and methods

### Animal husbandry and Pb^2+^ exposure paradigm

Adult female Long-Evans rats (225–250 g; Charles River Inc., Wilmington, MA, United States; RRID:RGD_2308852) were fed a diet containing either 0 or 1,500 parts per million (ppm) Pb^2+^-acetate (RMH 1000; Dyets, Bethlehem, PA, United States) for 10 days prior to breeding and throughout gestation and lactation. Litters were culled to 10 pups, weaned at twenty-one days of age (PN21) and maintained on the same diet as their respective dam to maintain a continuous Pb^2+^ exposure. At twenty-eight days (PN28), fifty days (PN50), or one hundred and twenty days after birth (PN120), male and female offspring were used for behavioral testing. For statistical purposes in each experiment, a single data point consisted of only one male or female pup per litter. Thus, the litter was the statistical unit. All animal studies were approved by the Florida International University Animal Care and Use Committee and have been carried out in accordance with the Guide for Care and Use of Laboratory Animals as stated by the U.S. National Institutes of Health.

### Blood Pb^2+^ levels

Rats were euthanized using CO_2_ and blood was collected transcardially and placed into 1 cc vacutainer EDTA tubes. Fifty μL of the blood sample was added to the LeadCare plus tubes and blood Pb^2+^ levels were measured 24–72 h after, using a Meridian Bioscience LeadCare plus analyzer following manufacturer’s instructions (Meridian Bioscience, Inc., Cincinnati, OH, United States).

### Drugs

Cocaine hydrochloride (Cocaine-HCl; Millipore Sigma, St. Louis, MO) was dissolved in sterile pharmaceutical-grade 0.9% saline to 5 or 15 mg mL^–1^ concentrations. Cocaine-HCl doses were delivered *via* intraperitoneal (IP) injections in a volume of 1 mL kg^–1^ body weight (bw) to yield doses of 5 or 15 mg kg^–1^. SCH23390 (Millipore Sigma, St. Louis, MO) was dissolved in sterile pharmaceutical-grade 0.9% saline solution to 0.05 and 0.1 mg mL^–1^ concentrations, administered *via* IP injections in a volume of 1 mL kg^–1^ bw to yield doses of 0.05 and 0.1 mg kg^–1^ bw. The rats were weighed the day the experimental procedures were performed.

### Locomotor activity

To determine if Pb^2+^ exposure increases the psychostimulant effects of an acute dose of cocaine at PN28, PN50, and PN120, naïve control animals or Pb^2+^-exposed male and female rats were placed into one of three experimental groups for the administration of vehicle-saline, 5 or 15 mg kg^–1^ cocaine-HCl. Prior to injection, all rats were placed in an automated activity chamber (Digiscan Animal Activity Monitor, Omnitech Electronics, Inc., Nova Scotia, Canada) and locomotor activity was recorded for 60 min to allow for behavioral habituation. After 60 min elapsed, rats were administered a single IP injection of vehicle-saline, 5 or 15 mg kg^–1^ cocaine-HCl before being placed back in the activity chamber for an additional 60 min to monitor locomotor activity. Total distance traveled (cm) was measured. Data are expressed as the percentage of the total distance traveled of the control-saline group within an age group. For subsequent experiments only the dose of cocaine that showed a significant locomotor activation in both experimental groups was used.

### Behavioral sensitization

Behavioral sensitization is defined as the augmented motor-stimulant response that occurs with repeated, intermittent exposure to a specific drug. Control and Pb^2+^ (1,500 ppm) PN28 and PN50 male or female rats were placed in the activity chamber for 60 min to record baseline activity. They were then given an i.p. injection of cocaine-HCl (15 mg kg^–1^), and allowed to explore an additional 60 min. This procedure took place for 5 consecutive days followed by 2 days of abstinence. On day 8 (reinstatement), animals were injected with 15 mg kg^–1^ of cocaine-HCl to determine behavioral sensitization. Total distance traveled was recorded and data are presented as the percentage of the total distance traveled on Day 1 for each treatment group.

### D1-dopamine receptor antagonist treatment

To determine the role of D1-dopamine receptor (D1R) on the effects of cocaine-induced locomotor sensitization, control, or Pb^2+^-exposed male and female rats were exposed to either vehicle or the selective D1R-antagonist, SCH-23390 (0.05 or 0.1 mg kg^–1^, IP) 15 min prior to the cocaine-HCl injection. At PN28, PN50, and PN120, animals were placed in an activity chamber and baseline locomotor activity was recorded for 60 min. Rats were then injected with either saline or SCH23390, and 15 min later injected with 15 mg kg^–1^ cocaine-HCl before being placed into the activity chamber for an additional 60 min. Total distance traveled (cm) was measured. Data are expressed as the percentage of inhibition when compared to the total distance traveled by the control group after receiving saline + 15 mg kg^–1^ cocaine-HCl.

### Tissue collection

Rat brains were harvested immediately after decapitation, snap-frozen, and then stored at −80^°^C until used. Fresh-frozen brains of control and Pb^2+^-exposed rats at PN14, PN28, PN50, and PN120 were sectioned at a 20-micron thickness in the coronal plane on a freezing cryostat (NX70, Epredia, Kalamazoo, MI) and thaw-mounted on poly-L-lysine-coated slides (Millipore Sigma, St. Louis, MO). Slides were stored at −80^°^C until used.

### Quantitative autoradiography

For D1R autoradiography, slides were pre-incubated in 50 mM Tris buffer (pH 7.4) at room temperature for 20 min. For total binding, slides were incubated in Tris buffer with [^3^H]-SCH23390 (1 nM; Perkin Elmer, Akron, OH) in Tris buffer (pH 7.4) for 30 min at room temperature. Non-specific binding was determined in adjacent slides by adding 5 μM butaclamol to the buffer. Slides were rinsed twice in buffer at 4^°^C and then dipped once in dH_2_O at 4^°^C. The slides were then dried at room temperature overnight.

After drying overnight, slides were apposed to Kodak Biomax MR film (MR-1, Millipore Sigma, St. Louis, MO), for 6 weeks. [^3^H]-Microscales (Amersham, Arlington Heights, IL) were included with each film to allow for quantitative analysis of images. Images were captured and analyzed using MCID Imaging software (InterFocus Imaging, Cambridgeshire, United Kingdom; MCID Core RRID:SCR_014419). A rat brain atlas (Paxinos and Watson, 1998) was used to define regions in the Olfactory Tubercle (OT), Nucleus Accumbens (NAC), Cerebral cortex, and Striatum (STR) to be analyzed. D1R levels in OT were evaluated by analyzing binding intensity measurements for anterior OT (aOT) (at Bregma 1.60 mm) and posterior OT (pOT) (at Bregma 0.70 mm). D1R levels in NAC were determined by analyzing binding intensity measurements for the NAC core (NACc) and NAC shell (NACs) (both at Bregma 1.60 mm). D1R in STR was determined by binding intensity measurements for rostral STR (rSTR) (at Bregma 1.60 mm), medial STR (mSTR) (at Bregma −0.26 mm), and caudal STR (cSTR) (at Bregma −0.92 mm). D1R binding in the anterior cingulate cortex (Acg), agranular region frontoparietal cortex (Agran), and the frontoparietal cortex motor area (FrPaM) were determined by binding measurement at bregma 1.60 mm.

### Statistical analysis

Statistical analyses were performed with SAS/STATv14.2 (SAS Institute Inc., Cary, NC; Statistical Analysis System RRID:SCR_008567) and GraphPad Prism 9 (GraphPad Software, San Diego, CA; GraphPad Prism RRID:SCR_002798). The outcome variable was examined for outliers and normality using a Kolmogorov-Smirnov test. Summary statistics including means, standard errors, frequencies, and proportions were generated for main outcome cocaine-induced locomotor activity, antagonist-dependent locomotor activity, and D1R levels and independent experimental conditions, treatment, sex, age, and brain region, respectively. Behavioral sensitization studies were analyzed using 2-way ANOVA with Repeated Measures, comparing Pb^2+^-treatment across days at each individual age and/or sex. Summary statistics for the D1R were further generated for all levels of independent experimental conditions. Univariate comparisons for D1R across experimental conditions consisted of *t*-test and ANOVA, depending on the number of categories in each condition, respectively. Sensitivity analysis was performed using the non-parametric equivalent tests, Wilcoxon Mann-Whitney U and Kruskal-Wallis test, respectively. To test all experimental conditions in one general linear model we applied linear regression starting with all four conditions. We tested two-way treatment interactions with age, sex, and brain region. Instances where interactions were significant warranted further stratified analysis of simplified models. All associations were considered significant at the alpha level of 0.05. All values are expressed as mean ± S.E.M.

## Results

### Animal weight and blood Pb^2+^ Levels

The Pb^2+^ exposure paradigm used in this study did not change the body weight gain of Pb^2+^-exposed rats at PN28, PN50, or PN120 (Albores-Garcia et al., 2021). BLL were significantly higher in both exposed groups in male and female rats, at all evaluated ages (Albores-Garcia et al., 2021).

### Locomotor activity

We have previously reported that Pb^2+^ exposure increases the cocaine-induced locomotor activity in PN50 male rats with BLL averaging 22 ± 0.7 μg/dL at PN50 (Stansfield et al., 2015). However, the possible role of Pb^2+^ exposure in regulating the sensitivity to cocaine-induced locomotor activity has not been assessed in younger or older animals nor in female rats. Here we show that chronic developmental Pb^2+^ exposure resulted in a statistically significant increase in the locomotor activity at PN28 and PN50 regardless of sex (Table 1). Table 1 shows the effect of Pb^2+^ exposure on cocaine-induced locomotor activation at different ages and the corrected pairwise comparisons by age, sex, and cocaine dose.

**TABLE 1:** Effect of Pb^2+^ exposure on the cocaine-induced locomotor activity in early adolescence (PN28), late adolescence (PN50), and adult rats (PN120).

Pb^2+^ effect on cocaine-induced locomotor activity			
Age	Pb^2+^ effect	Cocaine-dose	Sex
PN28	***F***_(1, 172)_ = **25.8, *p*** < **0.0001**	***F***_(2, 172)_ = **109.3, *p*** < **0.0001**	*F*_(1, 172)_ = 1.3, *p* = 0.25
PN50	***F***_(1, 196)_ = **14.45, *p* = 0.0002**	***F***_(2, 196)_ = **107, *p*** < **0.0001**	***F***_(1, 196)_ = **15.3, *p*** < **0.0001**
P120	*F*_(1, 183)_ = 2.6, *p* = 0.10	***F***_(2, 183)_ = **22.6, *p*** < **0.0001**	***F***_(1, 183)_ = **8.6, *p* = 0.004**
**Corrected pairwise comparisons by age and sex**			

	**Control vs. Pb^2+^ saline**	**Control vs. Pb^2+^ cocaine 5 mg kg^–1^**	**Control vs. Pb^2+^ cocaine 15 mg kg^–1^**

PN28-male	*F*_(1, 28)_ = 2.8, *p* = 0.10	*F*_(1, 30)_ = 1.4, *p* = 0.24	***F***_(1, 31)_ = **4.9, *p* = 0.034**
PN28-Female	***F***_(1, 26)_ = **5.8, *p* = 0.02**	***F***_(1, 26)_ = **14.5, *p* = 0.0008**	*F*_(1, 24)_ = 1.7, *p* = 0.20
PN50-male	*F*_(1, 30)_ = 1.1, *p* = 0.3	*F*_(1, 30)_ = 3.47, *p* = 0.07	***F***_(1, 30)_ = **5.8, *p* = 0.02**
PN50-Female	*F*_(1, 33)_ = 1, *p* = 0.32	***F***_(1, 31)_ = **6, *p* = 0.02**	*F*_(1, 35)_ = 1.6, *p* = 0.21
PN120-male	*F*_(1, 25)_ = 0, *p* = 0.97	*F*_(1, 29)_ = 0.04, *p* = 0.84	*F*_(1, 27)_ = 0.33, *p* = 0.57
PN120-female	*F*_(1, 33)_ = 0.6, *p* = 0.45	*F*_(1, 31)_ = 0.52, *p* = 0.47	*F*_(1, 31)_ = 1.96, *p* = 0.17

Bold values indicates significance at *p* < 0.05.

**FIGURE 1:**
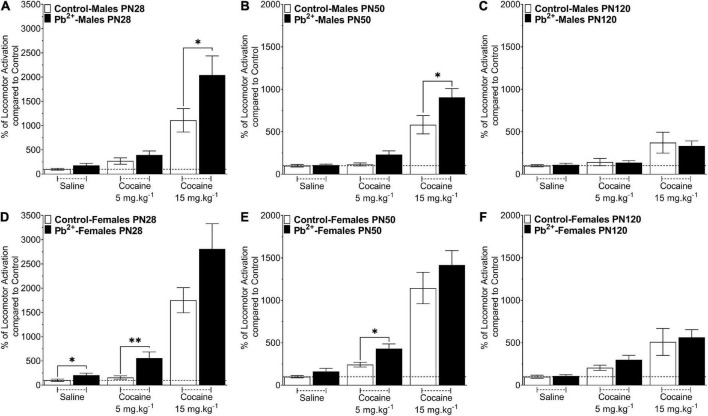
Effect of cocaine administration on the locomotor activity of control or Pb^2+^-exposed male **(A–C)** and female rats **(D–F)** at early-adolescence, postnatal day (PN) PN28 **(A,D)** late-adolescence PN50 **(B,E)** and adulthood **(C,F)**. Data are expressed as the percentage of locomotor activation compared to control-saline of the total distance traveled (cm) ± S.E.M for 60 min, immediately after the IP injection of Cocaine-HCl (0, 5 or 15 mg kg^– 1^). *n* = 11–21 animals/experimental group. *p < 0.05 relative to saline; ^**^p < 0.001 relative to saline.

Figure 1 shows the effects of Pb^2+^ exposure in the cocaine-induced locomotor activity in male and female rats at different ages. Early-adolescent (PN28) Pb^2+^-exposed rats had an overall higher response to cocaine-induced locomotor activity (Table 1). In early adolescent rats (PN28), we found a Pb^2+^ exposure effect on the locomotor activation (Table 1). In male rats, Pb^2+^ exposure did not affect the locomotor activity after saline or cocaine (5 mg kg^–1^) administration (Table 1 and Figure 1A), however a higher dose of cocaine (15 mg kg^–1^) induced an increase in the locomotor activity (Table 1 and Figure 1A). PN28 females showed a heightened sensitivity to Pb^2+^ exposure, showing an increase in locomotor activity in the absence of cocaine (a Pb^2+^ effect) and cocaine at 5 mg kg^–1^, but no changes after administering a higher dose of cocaine (15 mg kg^–1^) when compared to their control counterparts (Table 1 and Figure 1D).

In late adolescent rats (PN50) (Figures 1B,E), we observed cocaine-dose dependent effects in Pb^2+^ exposed rats compared to the control group (Table 1). More specifically, PN50 Pb^2+^-exposed male rats showed an increase in the cocaine-induced locomotor activity at the higher dose of cocaine (Table 1 and Figure 1B). PN50 female rats only showed an increase in the cocaine-induced locomotor activity at the lower cocaine dose (Table 1 and Figure 1E).

In adult (PN120) rats (Figures 1C,F), there was no effect of Pb^2+^ exposure (Table 1), as Pb^2+^ exposure did not cause an increase in locomotor activation in male or female rats at any of the cocaine doses administered (Table 1 and Figures 1C,F).

### Behavioral sensitization

**FIGURE 2:**
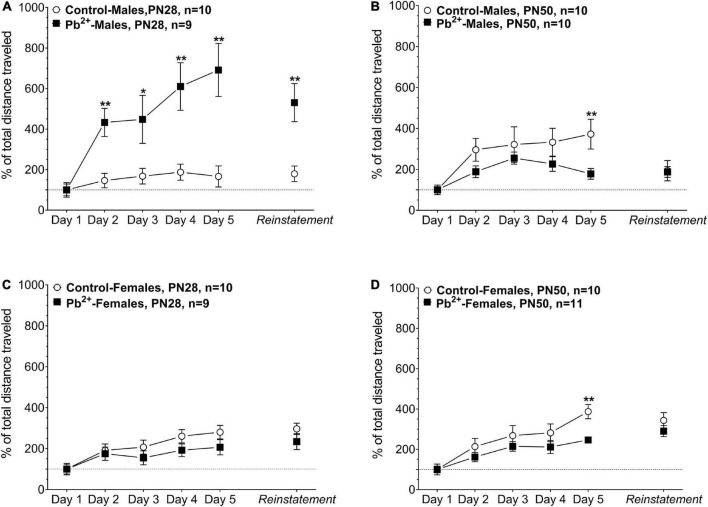
Effect of cocaine administration on the behavioral sensitization of control or Pb^2+^-exposed male **(A,B)** and female rats **(C,D)** at early-adolescence, postnatal day (PN) PN28 **(A,C)** late-adolescence PN50 **(B,D)**. Data are expressed as the percentage of locomotor activation compared to control-saline of the total distance traveled (cm) ± S.E.M. for 60 min, immediately after the IP injection of Cocaine-HCl (15 mg kg^– 1^). *n* = 9–11 animals/experimental group. *p < 0.05 relative to control; **p < 0.001 relative to control.

Chronic developmental Pb^2+^ exposure had a differential effect on male and female sensitization to cocaine. In early adolescence, Pb^2+^-exposed male rats showed a sustained exacerbation of the cocaine-induced locomotor activation during the sensitization phase of the protocol (days 1–5) suggesting a sustained disturbance of dopaminergic neurotransmission due to Pb^2+^ exposure. At reinstatement, PN28 Pb^2+^-exposed male rats exhibited a 300% increase in the cocaine-induced locomotor activation compared to their control counterparts (Figure 2 and Table 2). PN28 Pb^2+^-exposed females did not show any differences in behavioral sensitization (Figure 2 and Table 2). At PN50, male and female Pb^2+^-exposed rats showed an increase in the cocaine-induced locomotor activation on day 5 of the protocol, with no differences during reinstatement (Figure 2 and Table 2).

**TABLE 2:** Effect of Pb^2^^+^ exposure on the cocaine-induced behavioral sensitization in early (PN28) and late (PN50) adolescent rats.

Pb^2+^ effect on behavioral sensitization				
	PN28-males	PN50-males	PN28-females	PN50-females
Pb^2+^-effect	***F***_(1, 17)_ = **22.2, *p* = 0.0002**	*F*_(1, 18)_ = 2.7, *p* = 0.12	*F*_(1, 17)_ = 2.05, *p* = 0.17	*F*_(1, 19)_ = 3.94, *p* = 0.062
Day-effect	***F***_(5, 17)_ = **12.3, *p*** < **0.0001**	***F***_(5, 18)_ = **3.9, *p* = 0.014**	***F***_(5, 17)_ = **22, *p*** < **0.0001**	***F***_(5, 19)_ = **41.5, *p*** < **0.0001**
Pb^2+^ × time	***F***_(5, 17)_ = **7.1,** ***p*** = **0.001**	***F***_(5, 18)_ = **3.5, *p* = 0.023**	*F*_(5, 17)_ = 1.65, *p* = 0.20	***F***_(5, 19)_ = **3.75, *p* = 0.016**
Day 1	*p* = 1, *t* = 0	*p* = 1, *t* = 0	*p* = 1, *t* = 0	*p* = 1, *t* = 0
Day 2	***p*** = 0.0015, *t* = **-3.77**	*p* = 0.106, *t* = 1.7	*p* = 0.72, *t* = 0.36	*p* = 0.27, *t* = 1.14
Day 3	***p*** = 0.034, *t* = **-2.35**	*p* = 0.48, *t* = 0.73	*p* = 0.3, *t* = 1.1	*p* = 0.33, *t* = 0.99
Day 4	***p*** = 0.0024, *t* = **-3.57**	*p* = 0.18, *t* = 1.4	*p* = 0.15, *t* = 1.5	*p* = 0.20, *t* = 1.3
Day 5	***p*** = **0.0012, *t* = -3.88**	***p*** = **0.022, *t* = 2.5**	*p* = 0.16, *t* = 1.5	***p*** = **0.001, *t* = 3.9**
Reinstatement	***p*** = **0.0023, *t* = -3.6**	*p* = 0.91, *t* = 0.11	*p* = 0.20, *t* = 1.32	*p* = 0.26, *t* = 1.14

Bold values indicates significance at *p* < 0.05.

### Role of D1-dopamine receptor in cocaine-induced locomotor activity

We have previously reported that Pb^2+^-exposed PN50 male rats had a greater locomotor response to cocaine when compared to control animals (Stansfield et al., 2015) consistent with our present results (Figure 1). To show the role of the dopaminergic system in the cocaine-induced locomotor activation, we used different doses of the D1R antagonists SCH-23390. The data in Table 3 depicts the effect of the D1R antagonist on the percent inhibition of the cocaine-induced locomotor activation (15 mg kg^–1^), where saline + cocaine (15 mg kg^–1^) equals 0% inhibition (or 100% activation). Our data shows that the increased response in the cocaine-induced locomotor activity is completely blocked by the D1R antagonist SCH23390 in males and females at PN28 and PN120 relative to no antagonist (Table 3). At PN50, cocaine-induced locomotor activity is partially blocked by the D1R antagonist in males and completely blocked in females (Table 3).

**TABLE 3:** Effect of an antagonist of the dopamine-1 receptor (SCH23390) on the cocaine-induced locomotor activity of control or Pb^2+^-exposed male and female rats as a function of age.

% of inhibition of cocaine-induced locomotor activity					
	PN28				

	Males		Females		
	Control	Pb^2+^	Control	Pb^2+^	
**SCH23390 0.05 mg kg^–1^**	85.14 ± 4.94 *n* = 10	91.7 ± 3.5 *n* = 10	89.6 ± 5.5 *n* = 10	97.1 ± 0.7 *n* = 10	Treatment *F*_(1,115)_ = 2.26, *p* = 0.136 **Antagonist *F*_(2,115)_ = 63.8 *p*** < **0.0001** Sex *F*_(1,115)_ = 1, *p* = 0.30
**SCH23390 0.1 mg kg^–1^**	98.8 ± 0.35 *n* = 10	95.7 ± 2.7 *n* = 10	98.38 ± 0.6 *n* = 10	98.2 ± 0.5 *n* = 10	

	**PN50**				

**SCH23390 0.05 mg kg^–1^**	50.8 ± 12.34 *n* = 14	62.9 ± 9.9 *n* = 12	93.5 ± 2.3 *n* = 10	95.3 ± 1.7 *n* = 10	**Treatment *F*_(1,138)_ = 4.15, *p* = 0.043** **Antagonist *F*_(2,138)_ = 66.4, *p*** < **0.0001** Sex *F*_(1,138)_ = 0.25, *p* = 0.62
SCH23390 0.1 mg kg^–1^	86.4 ± 5 *n* = 14	89.3 ± 3.4 *n* = 12	98.4 ± 0.4 *n* = 12	96.1 ± 1.3 *n* = 10	

	**PN120**				

**SCH23390 0.05 mg kg^–1^**	92.2 ± 4 *n* = 8	80.35 ± 11.9 *n* = 8	95.4 ± 2.3 *n* = 10	95.8 ± 0.95 *n* = 10	Treatment *F*_(1,105)_ = 0.35, *p* = 0.55 **Antagonist *F*_(2,105)_ = 23.8, *p*** < **0.0001** Sex *F*_(1,105)_ = 1.8, *p* = 0.18
**SCH23390 0.1 mg kg^–1^**	90.2 ± 4.5 *n* = 8	89.1 ± 4.5 *n* = 10	98.2 ± 0.5 *n* = 10	97.4 ± 1 *n* = 10	

Data are expressed as the mean ± S.E.M of the percentage of inhibition of the cocaine-induced locomotor activity (control cocaine-HCl 15 mg kg**^–^**^1^ + saline equals 0% inhibition) the total distance traveled (cm) for 60 min after the administration (IP) of SCH23390 (0.05 or 0.1 mg kg**^–^**^1^) 15 min before Cocaine-HCl administration (15 mg kg**^–^**^1^). Bold values indicates significance at *p* < 0.05.

### D1-dopamine receptor quantitative autoradiography

**FIGURE 3:**
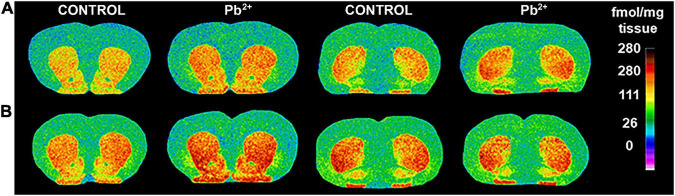
Representative images of [^3^H]-SCH23390 autoradiography binding to D1R in different brain regions of male **(A)** and female **(B)** control and Pb^2+^-exposed rats at postnatal day (PN) 28. The pseudocolor bar on the right side of each image indicates the level of specific receptor binding in the regions of interest (fmol/mg tissue).

To understand the underlying neurobiology of the exacerbated locomotor response to different doses of cocaine in Pb^2+^-exposed rats, we examined D1R levels in several brain regions implicated in reward and addiction (Figure 3). Given the increased susceptibility to Pb^2+^ neurotoxic effects at younger ages, we also examined D1R levels at PN14, a highly susceptible developmental age, at which it is difficult to assess the cocaine-induced locomotor effects.

**FIGURE 4:**
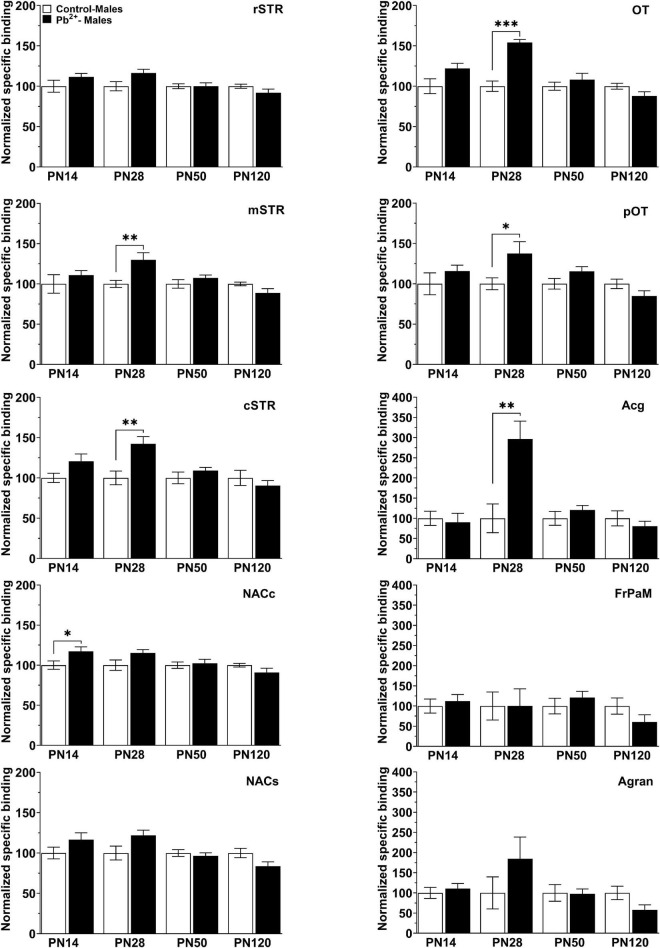
[^3^H]-SCH23390 specific binding to D1R in different brain regions in control and Pb^2+^-exposed male rats as a function of age. rstr, Rostral striatum; mSTR, medial striatum; cSTR, caudal striatum; NACc, nucleus accumbens core; NACs, nucleus accumbens shell; OT, olfactory tubercle; pOT, posterior olfactory tubercle; Acg, anterior cingulate cortex; Agran, agranular region frontoparietal cortex; FrPaM, frontoparietal cortex motor area. Values are mean ± S.E.M. *n* = 6–8 animals/experimental group. *p < 0.05 relative to control; ^**^p < 0.01 relative to control; ^***^p < 0.001 relative to control.

In males, Pb^2+^ exposure increased the levels of D1R in the NACc (Table 4 and Figure 4) at PN14. No other changes in D1R levels were detected at this age. At PN28, D1R levels of male rats were increased in the mSTR, cSTR, aOT, pOT, and Acg of Pb^2+^-exposed animals compared to controls (Table 4 and Figure 4). No changes were detected in the other brain regions evaluated at this age in Pb^2+^-exposed rats compared to controls. No changes in D1R levels in Pb^2+^-exposed animals vs. controls were detected in males at PN50 and PN120 (Table 4 and Figure 4).

**TABLE 4:** Summary statistics of [^3^H]-SCH23390 autoradiography in different brain regions of male and female rats as a function of age.

	PN14		PN28		PN50		PN120	
	Males	Females	Males	Females	Males	Females	Males	Females
rSTR	*F*_(1, 12)_ = 1.8, *p* = 0.21	*F*_(1, 12)_ = 4.2, *p* = 0.063	*F*_(1, 13)_ = 4.25, *p* = 0.06	***F***_(1, 12)_ = **17, *p* = 0.0014 ↑**	*F*_(1, 15)_ = 0, *p* = 0.98	*F*_(1, 10)_ = 0.9, *p* = 0.37	*F*_(1, 11)_ = 2.6, *p* = 0.14	*F*_(1, 10)_ = 0, *p* = 0.97
mSTR	*F*_(1, 12)_ = 0.75, *p* = 0.40	***F***_(1, 10)_ = **6.2, *p* = 0.032** **↑**	***F***_(1, 12)_ = **10.7, *p* = 0.007** **↑**	*F*_(1, 12)_ = 1.8, *p* = 0.20	*F*_(1, 15)_ = 1.4, *p* = 0.25	*F*_(1, 10)_ = 0, *p* = 0.98	*F*_(1, 11)_ = 4.2, *p* = 0.064	*F*_(1,_ **_9)_ = 6, *p* = 0.036** **↑**
cSTR	*F*_(1, 12)_ = 3.5, *p* = 0.086	*F*_(1, 12)_ = 2.6, *p* = 0.13	***F***_(1, 13)_ = **11.1, *p* = 0.005** **↑**	*F*_(1, 12)_ = 0, *p* = 0.99	*F*_(1, 15)_ = 1.3, *p* = 0.26	*F*_(1, 10)_ = 2.4, *p* = 0.15	*F*_(1, 11)_ = 0.7, *p* = 0.43	*F*_(1,_ **_10)_ = 9.25, *p* = 0.012** **↓**
NACc	***F***_(1, 12)_ = **4.9, *p* = 0.047** **↑**	*F*_(1, 12)_ = 1.3, *p* = 0.28	*F*_(1, 13)_ = 2.9, *p* = 0.11	***F***_(1, 12)_ = **7.4, *p* = 0.019** **↑**	*F*_(1, 15)_ = 0.12, *p* = 0.73	*F*_(1, 10)_ = 1, *p* = 0.33	*F*_(1, 11)_ = 2.7, *p* = 0.13	*F*_(1, 10)_ = 0.6, *p* = 0.45
NACs	*F*_(1, 12)_ = 2.1, *p* = 0.16	*F*_(1, 12)_ = 0.03, *p* = 0.86	*F*_(1, 13)_ = 3.5, *p* = 0.08	***F***_(1, 12)_ = **5.4, *p* = 0.038** **↑**	*F*_(1, 15)_ = 0.38, *p* = 0.54	*F*_(1, 10)_ = 3.6, *p* = 0.086	*F*_(1, 11)_ = 4.1, *p* = 0.06	***F***_(1, 10)_ = **10.5, *p* = 0.009** **↑**
OT	*F*_(1, 12)_ = 3.9, *p* = 0.07	*F*_(1, 12)_ = 0.99, *p* = 0.34	***F***_(1, 13)_ = **40, *p*** < **0.0001** **↑**	***F***_(1, 12)_ = **9.4, *p* = 0.0099** **↑**	*F*_(1, 15)_ = 0.7, *p* = 0.41	*F*_(1, 10)_ = 0.9, *p* = 0.39	*F*_(1, 11)_ = 3.8, *p* = 0.08	*F*_(1, 10)_ = 0.3, *p* = 0.6
pOT	*F*_(1, 12)_ = 1, *p* = 0.32	***F***_(1, 11)_ = **6.8, *p* = 0.024** **↑**	***F***_(1, 13)_ = **6.34, *p* = 0.026** **↑**	***F***_(1, 12)_ = **6.6, *p* = 0.024** **↑**	*F*_(1, 15)_ = 3.1, *p* = 0.09	*F*_(1, 10)_ = 3.2, *p* = 0.10	*F*_(1, 11)_ = 3, *p* = 0.11	*F*_(1, 10)_ = 0, *p* = 0.96
Acg	*F*_(1, 12)_ = 0.12, *p* = 0.73	*F*_(1, 12)_ = 0.34, *p* = 0.57	***F***_(1, 13)_ = **12, *p* = 0.004** **↑**	*F*_(1, 12)_ = 3.2, *p* = 0.09	*F*_(1, 15)_ = 1, *p* = 0.31	*F*_(1, 10)_ = 0.7, *p* = 0.42	*F*_(1, 11)_ = 0.7, *p* = 0.41	*F*_(1, 10)_ = 0.25, *p* = 0.62
FrPaM	*F*_(1, 12)_ = 0.26, *p* = 0.61	*F*_(1, 12)_ = 0.04, *p* = 0.85	*F*_(1, 13)_ = 0, *p* = 0.99	*F*_(1, 12)_ = 0.36, *p* = 0.56	*F*_(1, 15)_ = 0.7, *p* = 0.40	*F*_(1, 10)_ = 1.2, *p* = 0.3	*F*_(1, 11)_ = 2, *p* = 0.17	*F*_(1, 10)_ = 0.11, *p* = 0.74
Agran	*F*_(1, 12)_ = 0.35, *p* = 0.57	*F*_(1, 12)_ = 0.02, *p* = 0.9	*F*_(1, 13)_ = 1.66, *p* = 0.22	*F*_(1, 12)_ = 2.4, *p* = 0.15	*F*_(1, 15)_ = 0.01, *p* = 0.93	*F*_(1, 10)_ = 1.4, *p* = 0.27	*F*_(1, 11)_ = 3.8, *p* = 0.077	*F*_(1, 10)_ = 1.4, *p* = 0.27

Bold values indicates significance at *p* < 0.05.

**FIGURE 5:**
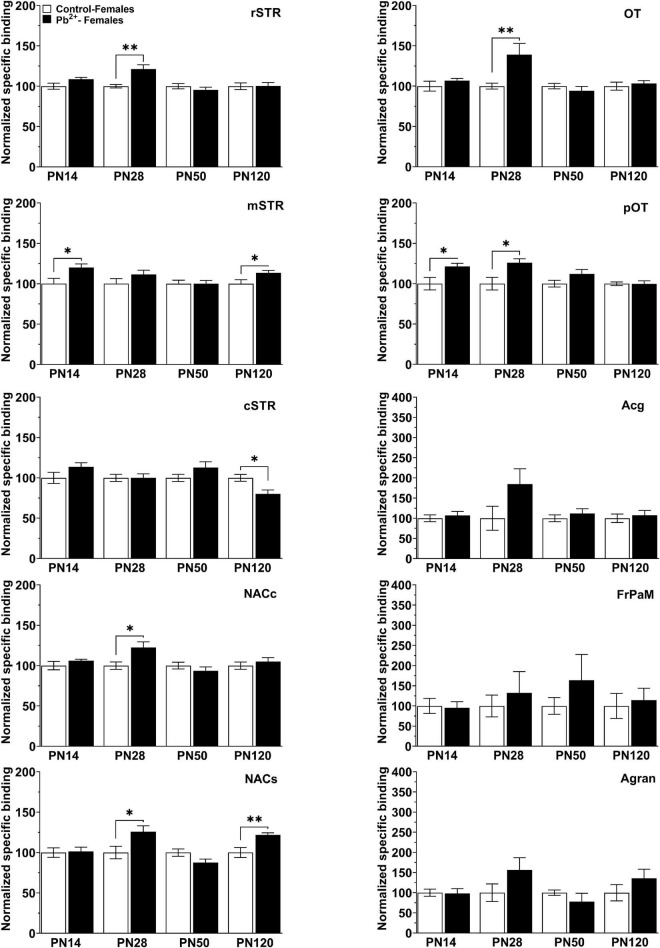
[^3^H]-SCH23390 specific binding to D1R in different brain regions in control and Pb^2+^-exposed female rats as a function of age. rstr, Rostral striatum; mSTR, medial striatum; cSTR, caudal striatum; NACc, nucleus accumbens core; NACs, nucleus accumbens shell; OT, olfactory tubercle; pOT, posterior olfactory tubercle; Acg, anterior cingulate cortex; Agran, agranular region frontoparietal cortex; FrPaM, frontoparietal cortex motor area. Statistical differences compared to their correspondent control group are indicated. Values are mean ± S.E.M. *n* = 6–8 animals/experimental group. *p < 0.05 relative to control; **p < 0.01 relative to control.

In females, Pb^2+^ exposure increased the levels of D1R in the mSTR and pOT (Table 4 and Figure 3) at PN14, with no other changes detected at this age. At PN28, D1R levels of female rats were increased in rSTR, NACc, NACs, aOT, and pOT (Table 4 and Figure 5). At PN50, Pb^2+^ exposure did not change the D1R levels of female rats in any of the brain regions evaluated (Table 4 and Figure 5). In PN120 female rats, there was an increase in the D1R levels of the mSTR and NACs of Pb^2+^-exposed rats, and a decrease of the D1R levels in the cSTR of Pb^2+^-exposed rats compared to controls (Table 4 and Figure 5).

## Discussion

The main finding of this study was that chronic developmental Pb^2+^ exposure exacerbates the acute psychostimulant effects of cocaine in adolescent male and female rats, but not in adult rats. The increased susceptibility to Pb^2+^ at earlier developmental stages is also supported at the receptor level, where we found that Pb^2+^ exposure significantly increased D1R levels in the STR, NAC core and shell, and the OT of male and female juvenile and early adolescent rats (PN14, PN28). It is of special interest that the key roles of these brain regions are associated with goal-directed behaviors, reward, and addiction.

Alterations in dopamine homeostasis have the ability to impact processes regulated by the dopaminergic system, such as motor activity (Centonze et al., 2003). Importantly, changes in dopaminergic transmission could alter dopaminergic circuits underlying reward, motivation, and executive function which could modify the motivational value of a drug and contribute to an increased risk of substance use disorder (Baler and Volkow, 2006; Volkow et al., 2011, 2012). Further studies are needed to understand how Pb^2+^ exposure could modify these circuits.

Dopamine regulates molecular signaling through the STR, the NAC and the OT, and the downstream effects of dopamine are dependent on the activation of D1 and D2 dopaminergic receptors subtypes. Activation of either opposing pathway regulates many central nervous system processes, which are susceptible to the neurotoxic effects of Pb^2+^ exposure (Cory-Slechta and Widzowski, 1991; Nation et al., 2000; Gedeon et al., 2001; Szczerbak et al., 2007). For example, previous work from our laboratory has shown that Pb^2+^ exposure increases dopamine turnover and dopamine-2 receptor levels in the STR of Pb^2+^-exposed PN50 male rats (Stansfield et al., 2015).

To the best of our knowledge, this is the first study to describe significant increases in D1R in the OT of Pb^2+^-exposed male and female juvenile and adolescent rats. Furthermore, our results demonstrate that D1R levels in the OT are markedly elevated by Pb^2+^ exposure relative to the STR and NAC. This is important and relevant because the OT has been identified as a trigger zone, acting as a synaptic junction in the mesocorticolimbic system where the circuitry underlying reward function is first activated (Ikemoto, 2010). The OT serves as a sensory integration site where the auditory, visual, gustatory, and olfactory cues that lead to a reward response first converge (Chiang and Strowbridge, 2007; Wesson and Wilson, 2010; Wesson, 2020), hence its activation by psychostimulant drugs may be a central component triggering circuitry underlying drug-related reward (Ikemoto, 2010).

Experimental evidence suggests an increased sensitivity to drugs in the OT (Ikemoto, 2003, 2007, 2010). For example, studies have shown an increased rate of self-administration in psychostimulant treated rats when injected in the OT (Ikemoto, 2003, 2010). These studies suggest that neurotransmission disturbances in the OT could play a significant role in the dopaminergic system dysregulation that underlies reward, drug use and abuse (Volkow and Li, 2005; Volkow et al., 2011, 2012; Wesson, 2020). However, the potential role of the OT as the mediator of Pb^2+^-induced behavioral changes needs investigation.

We found an exacerbated and sustained increase in the psychostimulant effects of cocaine in Pb^2+^-exposed early-adolescent male rats, reinforcing our hypothesis that Pb^2+^ exposure effects are more pronounced at early developmental stages and may play a role in addictive behaviors. Our results also suggest that Pb^2+^ exposure produces a sex-dependent susceptibility to cocaine, as Pb^2+^-exposed females exhibited a heightened locomotor response to a lower dose of cocaine (5 mg kg^–1^), when compared to Pb^2+^-exposed males (15 mg kg^–1^). These sex-dependent differences could be due to estrous cycle-dependent variations in progesterone and estrogen levels, as it has been shown that fluctuations in hormonal levels alters dopaminergic transmission (Becker and Beer, 1986; Bobzean et al., 2014).

Behavioral sensitization has a strong dopaminergic component, shown by a prevention of sensitization after the administration of the D1R antagonist SCH23390 on the reinstatement day (McDougall et al., 2017). It has been suggested that the behavioral sensitization protocol could assess the relapse rate based on the strength of withdrawal, aiming that those individuals that experienced more physical and emotional adverse effects related to drug withdrawal are more susceptible to relapse. Future studies are necessary to elucidate if the changes in D1R levels induced by Pb^2+^ exposure are further manifested in other stages of addiction and to assess if other neurotransmitter systems are affected, as well as to evaluate the consequences of Pb^2+^ exposure on acquisition, maintenance, escalation, withdrawal, and relapse. Our work shows that Pb^2+^ exposure increases the locomotor response to different doses of cocaine, however, it does not explore the possible role of Pb^2+^ exposure on self-administration and drug relapse, aspects of drug addiction that should be further explored.

The role of dopaminergic signaling on cocaine-induced locomotor activity and the heightened response of Pb^2+^-exposed animals suggests a potential role of increased D1R levels in the ventral STR as a mediator of the cocaine-induced locomotor activity in Pb^2+^-exposed animals. There are other neurotransmitter systems involved in SZ that have been previously shown to be affected by Pb^2+^ exposure, and dopaminergic signaling could be modulated by these other systems. For example, opioid receptors are highly implicated in reward and therefore a key signaling system in drug abuse mechanisms (Mulder et al., 1984; Spanagel et al., 1992; Shippenberg and Rea, 1997; Karkhanis et al., 2017), as well as a potential target of Pb^2+^ exposure (Albores-Garcia et al., 2021), particularly in early developmental stages. Dopamine receptors can form heteromers with opioid receptors, D2 dopamine receptors, and NMDA receptors, among others (reviewed in Derouiche and Massotte, 2019), and they can be involved in substance use disorder. Our data shows that Pb^2+^ exposure alters D1R levels in several brain regions implicated in substance abuse and previous studies from our group show that Pb^2+^ exposure affects other neurotransmission systems, which could translate at the behavioral level (Guilarte and McGlothan, 2003; Neal et al., 2010, 2011; Stansfield et al., 2015; Albores-Garcia et al., 2021). Additionally, other studies have shown the role of environmental toxin exposures on drug sensitivity. For example, developmental exposure to polychlorinated biphenyls (PCBs) has been shown to increase early cocaine-drug seeking on male rats (Miller et al., 2017a) and enhances behavioral sensitization to cocaine (Miller et al., 2017b).

The SZ brain is in a state of endogenous sensitization, where a heightened behavioral response and augmented dopamine release are observed after drug administration or exposure to stress. Animal models of SZ suppose that vulnerable neuronal circuits endure gradual changes during early development leading to psychosis. Preclinical and human studies have tried to characterize sensitization mechanisms and their link with psychosis. Our data suggest an increased susceptibility to drug sensitization in Pb^2+^-exposed animals, particularly at younger ages. Supplementary studies need to assess the potential of Pb^2+^ exposure on molecular and epigenetic targets associated with dopamine signaling and neuroplasticity.

This study presents some limitations, as it can neither prove causal relationships nor draw conclusions regarding molecular mechanisms underlying the observed behavioral associations. However, our results suggest a putative role of dopaminergic disturbances in Pb^2+^-exposed animals that needs to be further explored in order to explain the observed behavioral outcomes. In addition, this work is part of a long-standing project with data suggesting that exposure to environmental toxins during critical stages of brain development may contribute to the etiology of mental disorders (Abazyan et al., 2014; Stansfield et al., 2015), such as SZ and an increased susceptibility to substance use disorder (Albores-Garcia et al., 2021).

Taken together, our results support the hypothesis that chronic early life Pb^2+^ exposure increases sensitivity to the psychostimulant effects of cocaine by inducing a hyperactive dopaminergic state reflected by increase D1R levels in the OT, NAC and STR of young male and female rats. In addition, alterations in dopaminergic neurotransmission play a fundamental role in the increased susceptibility to drug addiction onset, and the developing brain is particularly sensitive to the detrimental effects of drug-induced neurotransmission dysregulation. As a consequence, alterations in the neurocircuitry resulting from drug use in adolescence can increase the susceptibility to drug use at a later age (Caffino et al., 2021).

## Conclusion

Our results suggest that early life Pb^2+^ exposure sensitizes adolescent rats to the psychostimulant effects of cocaine in a rat model that recapitulates SZ-like pathophysiology. These findings are relevant in the public health context because Pb^2+^ exposure, drug abuse, and psychiatric disorders are of major health concern in the United States. It should be noted that etiological factors in the increased prevalence of drug abuse and psychiatric disorders have overlook the role of exposure to ubiquitous environmental neurotoxicants. In summary, this study suggests Pb^2+^ exposure as a risk factor for psychiatric disorders and substance abuse.

## Data availability statement

The raw data supporting the conclusions of this article will be made available by the authors, without undue reservation.

## Ethics statement

The animal study was reviewed and approved by the Florida International University Institutional Animal Care and Use Committee.

## Author contributions

TG, DA-G, KS, and JM were responsible for the conception of the study, design of experiments, and interpretation of the data. DA-G, KS, and JM acquired the data. DA-G, KS, JM, and ZB analyzed the data. TG, DA-G, and ZB wrote the manuscript. JM and KS commented on the manuscript. All authors contributed to the manuscript and approved the submitted version.

## Abbreviations

Acg: anterior cingulate cortex
Agran: agranular region frontoparietal cortex
aOT: anterior olfactory tubercle
cSTR: caudal striatum
D1R: Dopamine-1 receptor
D2R: Dopamine-2 receptor
FrPaM: frontoparietal cortex motor area
OT: olfactory tubercle
Pb^2+^: Lead
PN: Postnatal
pOT: posterior olfactory tubercle
STR: Striatum
mSTR: medial striatum
rSTR: rostral striatum.
